# Association of Stroke Impairment Assessment Set and Dysphagia in the 1st Week after Acute Ischemic Stroke with Early Discharge to Home Following Endovascular Therapy: A Retrospective Observational Study

**DOI:** 10.1298/ptr.E10313

**Published:** 2024-12-24

**Authors:** Eisei HARAYAMA, Shota TANAKA, Kota YAMAUCHI, Shuji ARAKAWA

**Affiliations:** 1 Department of Rehabilitation, Social Medical Corporation Steel Memorial Yawata Hospital, Japan; 2 Department of Behavior and Health Sciences, Graduate School of Human-Environment Studies, Kyushu University, Japan; 3 Department of Stroke and Neurological Center, Steel Memorial Yawata Hospital, Japan

**Keywords:** EVT, Ischemic stroke, Physical therapy, SIAS, Dysphagia

## Abstract

Objective: In this study, we aimed to identify factors associated with the likelihood of early discharge to home by the 7th-day assessment in patients with ischemic stroke who underwent endovascular therapy (EVT). Methods: Among the 128 patients with ischemic stroke who underwent EVT, 2 groups were identified: an early discharge to the home group and the transfer group, with patients included in the latter group needing to be transferred to rehabilitation hospitals. Variables from the 7th day were used as explanatory variables to determine the outcome. Multiple logistic regression analysis was conducted using these variables. Receiver operating characteristic (ROC) curves for the significant factors were obtained, and cutoff values were calculated. Results: There were 19 patients (14.8%) in the early discharge to the home group and 109 patients (85.2%) in the transfer group. The Stroke Impairment Assessment Set (SIAS) (odds ratio [OR]: 1.16, 95% confidence interval [CI]: 1.02–1.32, p = 0.03) and the presence of dysphagia (OR: 8.75, 95% CI: 1.55–49.45, p = 0.01) were significant factors associated with early discharge home. The area under the ROC curve of the SIAS was 0.90, with a cutoff value of 63.5 points. Conclusion: Our results suggest that SIAS scores and the presence of dysphagia within the 1st week post-onset are significant predictors of whether patients with ischemic stroke who undergo EVT can be early discharged home.

## Introduction

In recent years, recombinant tissue-plasminogen activator (rt-PA) and endovascular therapy (EVT) have been used to treat acute ischemic stroke (AIS) in the hyperacute phase. EVT has been increasingly adopted, resulting in a growing number of patients undergoing rehabilitation. Clinical trials of EVT for main artery occlusion in the anterior circulation system have shown a significant increase in the number of cases with satisfactory outcomes after 90 days, demonstrating the safety and effectiveness of the treatment^1–6)^. Additionally, the treatment window for cerebral infarctions has been expanded to exceed 6 h after onset^7,8)^. Recent reports also include cases of posterior circulatory system obstruction^9)^. Early rehabilitation within 48 h after EVT is associated with fewer complications and better outcomes in activities of daily living compared to the corresponding presented in cases initiating rehabilitation after 48 h^10)^.

In rehabilitation following EVT, attention must be paid to treatment-specific complications, such as symptomatic intracerebral hemorrhage (sICH), which occurs in approximately 5% of cases compared to the medical treatment group. ICH is a hemorrhagic complication that may occur after EVT, including small punctate hemorrhagic infarcts and extensive brain parenchymal hemorrhage. Of these, sICH corresponds to extensive brain parenchymal hemorrhage after EVT^11)^. Despite the benefits of EVT over conventional medical treatment, predicting outcomes remains challenging due to a subset of patients experiencing poor outcomes. The Highly Effective Reperfusion Evaluation in Multiple Endovascular Stroke Trials^6)^ reported that 21.5% of cases had poor outcomes with a modified Rankin Scale (mRS) score of ≥5 points.

In the treatment of cerebral infarction, the framework for the medical and nursing care provision system has been clarified, and collaboration between acute phase hospitals requiring intensive treatment, convalescent phase rehabilitation hospitals primarily providing rehabilitation, and other facilities is becoming increasingly important. Acute care hospitals face the necessity of shortening the length of stay, necessitating immediate transfers after symptom onset. Chevalley et al.^12)^ reported that motor function at admission is a critical factor for returning home in acute stroke rehabilitation. Treatment outcomes for EVT are typically measured using mRS after 90 days, the National Institutes of Health Stroke Scale (NIHSS), and the modified Thrombolysis in Cerebral Infarction (mTICI)^13)^, which is an indicator of recanalization of occluded blood vessels. There are few research reports evaluating physical function and predicting outcomes in patients with AIS after EVT, and this remains unclear. Therefore, research predicting outcomes using physical therapy assessment indices would be useful. Therefore, we focused on the Stroke Impairment Assessment Set (SIAS)^14)^. The SIAS is particularly recommended by the Japanese Guideline for the Management of Stroke^15)^. A study using SIAS total scores to predict outcomes in patients with stroke^16)^ indicated that SIAS scores have predictive validity and are useful for predicting outcomes. Thus, determining whether a patient can return home within the limited hospitalization period is crucial.

We hypothesized that SIAS, an assessment of physical function, is associated with the outcome of AIS after EVT and may predict the outcome. We utilized the SIAS, a comprehensive tool for assessing functional impairment, alongside other assessment methods, to predict patient prognosis. In this study, we aimed to identify factors associated with the ability of patients with AIS who underwent EVT to be early discharged home, based on a 7-day assessment.

## Methods

### Study design and patients

This was a single-center retrospective observational study. The study period was from January 2016 to August 2023. This study was conducted at Steel Memorial Yawata Hospital.

The participants were patients with AIS admitted to our hospital who underwent EVT. EVT treatment was indicated based on the physician’s diagnosis, and rt-PA therapy was administered to eligible patients deemed suitable for treatment. The inclusion criteria were as follows: (1) age ≥18 years, (2) EVT for cerebral infarction, (3) previous rehabilitation, and (4) pre-symptomatic mRS ≤3. The exclusion criteria were as follows: (1) mRS ≥4 before onset, (2) death during hospitalization, and (3) cases without rehabilitation. The mRS^17)^ is an index that assesses social disadvantage and behavioral restrictions on a 7-point scale from Grade 0 (asymptomatic) to Grade 6 (death). Individuals with presymptomatic mRS scores ≥4 points were excluded due to the significant impact on functional prognosis.

### Outcome

Regarding the outcome of discharge destination, we classified patients based on Schrage et al.’s classification^18)^. Patients who were discharged to their pre-hospital living environment or an independent elderly facility were categorized as the early home discharge group. Those who were discharged to a convalescent hospital or nursing facility were categorized as the transfer group.

### Assessments

We collected basic demographic and clinical attributes, including age, sex, body mass index, type of stroke, site of occlusion, mTICI score, and the presence or absence of hypertension, dyslipidemia, diabetes, atrial fibrillation, and chronic kidney disease—known risk factors for cerebral infarction. Effective recanalization was defined as mTICI ≥2b^6)^, an index of recanalization success post-EVT. Additional clinical assessments included pre-admission mRS, time to start treatment (min), treatment details (including rt-PA administration), NIHSS at onset, presence or absence of sICH and other complications (such as respiratory and urinary tract infections, or worsening heart failure), length of stay (LOS), discharge outcomes, SIAS scores, presence of higher brain dysfunction, Functional Ambulation Categories (FAC), and the Functional Oral Intake Scale (FOIS). In general, there is no set protocol for physical therapy after EVT. After surgery, a physician must determine whether there are any problems with the treatment of the treatment puncture site and whether or not sICH is present. In risk management, the intervention is started after confirming the presence or absence of sICH with a head CT scan the day after surgery, and the timing of intervention varies from case to case. For these reasons, all assessments were performed 1 week post-treatment.

The mRS and NIHSS scores were assessed by physicians at admission. The NIHSS, consisting of 11 categories covering 15 neurological examination items, scores each item from 0 to 4 points, with a total possible score ranging from 0 to 42 points; higher scores indicate greater severity^19)^. Physicians diagnosed sICH and other complications during hospitalization. A physical therapist assessed functional impairment using the SIAS^16,20)^ within 7 days of onset. The SIAS assesses motor function on the paralyzed side (0–5 points), muscle tone, sensation, joint range of motion, pain, trunk function, higher brain function, and non-paralyzed side function (each 0–3 points). Higher brain dysfunction in SIAS includes unilateral spatial neglect (USN) and aphasia, with attention disorders assessed by physicians and occupational therapists. FAC, an index that assesses walking independence in 6 categories^21)^, defined practical walking ability as FAC ≥3. FOIS^22)^ assessed swallowing function on a 7-point scale from level 1 (no oral intake) to level 7 (normal swallowing function), with levels 6 and 7 indicating no dysphagia and level ≤5 indicating dysphagia^23)^. Social information included the presence of a cohabiting family member and pre-hospitalization certification by the long-term care insurance system^24)^. Japan's public long-term care insurance service certifies individuals at 7 levels of care after professional assessment.

### Ethics

This research was conducted in accordance with the Declaration of Helsinki. The purpose of the research and ethical considerations were fully explained to the participants, and their consent was obtained to protect their personal information. This study was performed after approval by the Ethics Committee at Steel Memorial Yawata Hospital (approval number: 23-70).

### Sample size calculation

To our knowledge, there are no previous studies investigating factors associated with early home discharge in patients with AIS after EVT. Therefore, we calculated the sample size as if there were no previous studies. The sample sizes were calculated using G*Power 3.1.9.7, a 2-tailed test with α = 0.05, power (1 – β) of 0.8, and an effect size of 0.5. Consequently, a sample size of at least 128 participants was required.

### Statistical analysis

The Shapiro–Wilk test was used to examine the normality of all variables. Comparisons between the 2 groups were performed using the unpaired t-test for normally distributed continuous variables, the χ^2^ test for categorical variables, and the Mann–Whitney U test for non-normal continuous variables and ordinal scale variables. For multivariate analysis, items that showed significant differences in univariate analysis and items that were found to be related to the outcome from previous studies were used as explanatory variables, and a logistic regression analysis was performed using discharge outcome as the objective variable. Model 1 included predictor variables related to basic attributes, clinical assessment, and social information. In Model 2, age and time to treatment initiation were forcibly included as covariates to adjust for potential confounding, irrespective of the univariate analysis. The covariates included in the logistic regression analysis were tested for multicollinearity using Spearman’s rank correlation coefficient and the variance inflation factor (VIF) for each independent variable. Variables with an absolute value of correlation coefficient of ≥0.8 or VIF >7 were considered clinically meaningful and selected for inclusion. For continuous variables identified through multiple logistic regression, receiver operating characteristic (ROC) curves were obtained, and cutoff values were calculated using Youden’s Index method. Statistical analysis was performed using SPSS for version 23 (IBM Japan, Tokyo, Japan), with the significance level set at 5%.

## Results

### Number of participants assigned to early home discharge groups and transfer groups

**Fig. 1.**
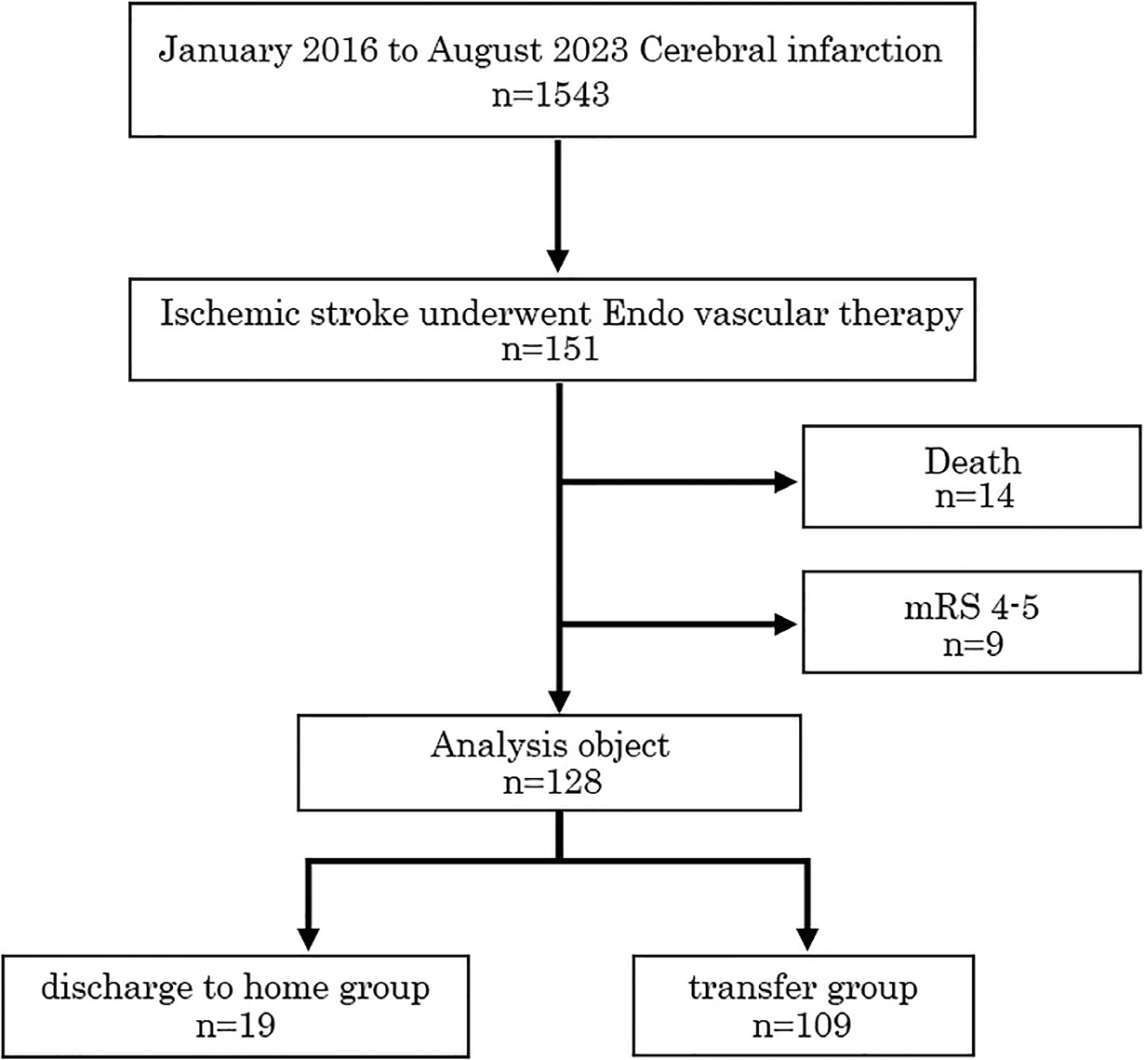
Flowchart of this study

The flow chart of the participants is shown in Figure 1. During the study period from January 2016 to August 2023, 1543 patients with AIS were admitted to our hospital. Of these, 151 patients who underwent EVT were included in the study. Fourteen patients who died in the hospital and 9 patients with a prehospital mRS score of 4–5 points were excluded. Therefore, a total of 128 patients were included in the analysis, comprising 19 (14.8%) in the early home discharge group and 109 (85.2%) in the transfer group.

### Comparison of early home discharge groups and transfer groups

Among the cases who underwent EVT for AIS, 14.8% were early discharged home, while 85.2% were transferred to rehabilitation hospitals. The comparison results of basic attributes, clinical assessments, and social information are shown in Tables 1 and 2. The SIAS was significantly higher in the early home discharge group compared to that in the transfer group (71.4 ± 5.4 vs. 40.8 ± 23.7, p <0.001). Significant differences were observed in the type of cerebral infarction (p <0.001) and site of occlusion, but no significant differences were noted in age or sex (Table 1). In terms of clinical assessments (Table 2), the transfer group had higher NIHSS scores (p <0.001), a lower effective recanalization rate (p = 0.03), more cases of pneumonia (p = 0.003), and more instances of swallowing disorders (p <0.001) compared to those of the home discharge group. Additionally, the transfer group had more cases requiring assistance with walking (p <0.001) and longer LOS (p = 0.003). Regarding social information, there were no significant differences in whether patients lived alone or had long-term care insurance (Table 2).

**Table 1.** Comparison of basic attributes between early home discharge group and transfer hospital group

	Early home discharge (n = 19)	Transfer to hospital (n = 109)	p-Value
Age, years^†^	73.9 (±13.7)	79.9 (±9.3)	0.07
Male sex (%)*	11 (57.9)	50 (45.9)	0.33
BMI, kg/m^2†^	23.9 (±4.5)	21.9 (±3.9)	0.06
Ischemic stroke (%)			
Cardiogenic cerebral embolism*	13 (68.4)	74 (67.9)	<0.01
Atherothrombotic*	0 (0)	8 (7.3)	
Embolic cerebral infarction*	6 (31.6)	21 (19.3)	
Thrombosis of unknown*	0 (0)	6 (5.5)	
Closed area (%)			
ICA*	1 (5.3)	27 (24.8)	0.05
ACA*	0 (0)	4 (3.7)	0.52
MCA M1*	10 (52.6)	41 (37.6)	<0.001
MCA M2*	4 (21.1)	22 (20.2)	
MCA M3*	1 (5.3)	4 (3.7)	
PCA*	2 (10.5)	1 (0.9)	0.06
VA*	1 (5.3)	5 (4.6)	0.63
BA*	0 (0)	5 (4.6)	0.44
Risk factors (%)			
Hypertension*	11 (57.9)	73 (67.0)	0.44
Atrial fibrillation*	13 (68.4)	81 (74.3)	0.59
Diabetes mellitus*	7 (36.8)	37 (33.9)	0.81
Dyslipidemia*	7 (36.8)	41 (37.6)	0.95
Chronic kidney disease*	4 (21.1)	18 (16.5)	0.42

*Chi-squared test; ^†^Mann–Whitney *U* test

Variables are expressed as mean (± standard deviation), median (interquartile range), or number of patients (%).

BMI, body mass index; ICA, internal carotid artery; ACA, anterior cerebral artery; MCA, middle cerebral artery; PCA, posterior cerebral artery; VA, vertebral artery; BA, basilar artery

**Table 2.** Comparison of clinical assessment and social information between early home discharge group and transfer group

	Early home discharge (n = 19)	Transfer to hospital (n = 109)	p-Value
mRS, points^†^	0 (0–2)	1 (0–2)	0.47
NIHSS, score^†^	8 (6–12)	18 (12–23)	<0.001
0–7	6	11	
8–16	10	30	
>16	3	68	
rt-PA treatment (%)*	12 (63.2)	62 (56.9)	0.61
mTICI ≥ 2b (%)*	18 (94.7)	80 (73.4)	0.03
Time to treatment initiation, min^†^	250.4 (±132.5)	167.5 (±93.4)	0.06
Postoperative complications (%)			
sICH*	0 (0)	11 (10.1)	0.16
Urinary tract infection*	0 (0)	17 (15.6)	0.05
Pneumonia*	0 (0)	32 (29.4)	0.003
Cardiac insufficiency*	0 (0)	8 (7.3)	0.27
Dysphagia (FOIS ≤ 5) (%)*	2 (10.5)	91 (83.5)	<0.001
0–5	2	91	
6–7	17	18	
Attention disorder (%)*	3 (15.8)	17 (15.6)	0.61
USN (%)*	0 (0)	51 (46.8)	<0.001
Aphasia (%)*	3 (15.8)	61 (56.0)	0.001
SIAS, points^†^	71.4 (±5.4)	40.8 (±23.7)	<0.001
FAC, categories^†^	3 (3–4)	1 (0–2)	<0.001
LOS, days^†^	25.5 (±12.7)	36.9 (±15.4)	0.003
Living alone (%)*	0 (0)	13 (11.9)	0.11
Long-term care insurance (%)*	6 (32)	35 (32)	0.96

*Chi-squared test; ^†^Mann–Whitney *U* test.

Variables are expressed as mean (± standard deviation), median (interquartile range), or number of patients (%).

mRS, modified Rankin Scale; NIHSS, National Institutes of Health Stroke Scale; rt-PA, recombinant tissue–plasminogen activator; mTICI, modified Thrombolysis in Cerebral Infraction; sICH, symptomatic intracerebral hemorrhage; FOIS, Functional Oral Intake Scale; USN, unilateral spatial neglect; SIAS, Stroke Impairment Assessment Set; FAC, Functional Ambulation Categories; LOS, length of stay

### Correlation and VIF of each variable

Spearman’s rank correlation coefficient and VIF between variables with significant differences are shown in Table 3. There was a significant positive correlation (r = 0.82) between SIAS and FAC. The VIF was <7 for all variables.

**Table 3.** Spearman’s correlation coefficient for covariates between early home discharge group and transfer group

	Age	NIHSS	SIAS	FAC	Time to treatment initiation	VIF
Age	1					1.07
NIHSS	0.07	1				1.32
SIAS	–0.20*	–0.46**	1			1.94
FAC	0.21*	0.37**	–0.65**	1		1.78
Time to treatment initiation	–0.11	–0.17	0.05	–0.09	1	1.05

*p< 0.05; **p< 0.01.

NIHSS, National Institutes of Health Stroke Scale; SIAS, Stroke Impairment Assessment Set; FAC, Functional Ambulation Categories; VIF, variance inflation factor

### Predictors of home discharge after EVT

SIAS and FAC were strongly correlated (r = 0.82). Therefore, logistic regression analysis was performed with SIAS as an explanatory variable (Table 4). The objective variable was defined as the presence or absence of transfer to another hospitals. In Model 1, the explanatory variables included NIHSS, SIAS, the presence or absence of effective recanalization, dysphagia, and time to treatment initiation. The analysis identified 2 significant factors: SIAS (odds ratio [OR]: 1.16, 95% confidence interval [CI]: 1.02–1.32, p = 0.03) and dysphagia (OR: 8.75, 95% CI: 1.55–49.45, p = 0.01). Model 2 adjusted for potential confounding and included NIHSS, SIAS, the presence or absence of effective recanalization, dysphagia, and time to treatment initiation, MCA, and age as explanatory variables. After adjusting for age, SIAS remained predictive (OR: 1.24, 95% CI: 1.04–1.50, p = 0.02) along with dysphagia (OR: 8.35, 95% CI: 1.34–52.07, p = 0.02). The predictive value was 91.4% (Table 4).

**Table 4.** Logistic regression analysis between early home discharge group and transfer group

	Model 1		Model 2	
Predictor	OR (95% CI)	p-Value	OR (95% CI)	p-Value
NIHSS	0.92 (0.81–1.03)	0.15	0.92 (0.81–1.05)	0.22
SIAS	1.16 (1.02–1.32)	0.03*	1.24 (1.04–1.50)	0.02*
mTICI	0.52 (0.04–7.18)	0.63	0.29 (0.01–6.67)	0.44
Dysphagia (FOIS ≤ 5)	8.75 (1.55–49.54)	0.01*	8.35 (1.34–52.07)	0.02*
Time to treatment initiation	1.01 (1.00–1.01)	0.13	1.01 (1.00–1.01)	0.14
MCA	–	–	0.59 (0.28–1.23)	0.16
Age	–	–	1.03 (0.96–1.11)	0.42

*p <0.05.

Hosmer–Lemeshow test: Model 1, p = 0.852, Model 2, p = 0.999.

Predictive value: Model 1, 93.0%, Model 2, 91.4%.

OR, odds ratio; CI, confidence interval; NIHSS, National Institutes of Health Stroke Scale; SIAS, Stroke Impairment Assessment Set; mTICI, modified Thrombolysis in Cerebral Infraction; FOIS, Functional Oral Intake Scale; MCA, middle cerebral artery

### ROC curve for home discharge prediction

**Fig. 2.**
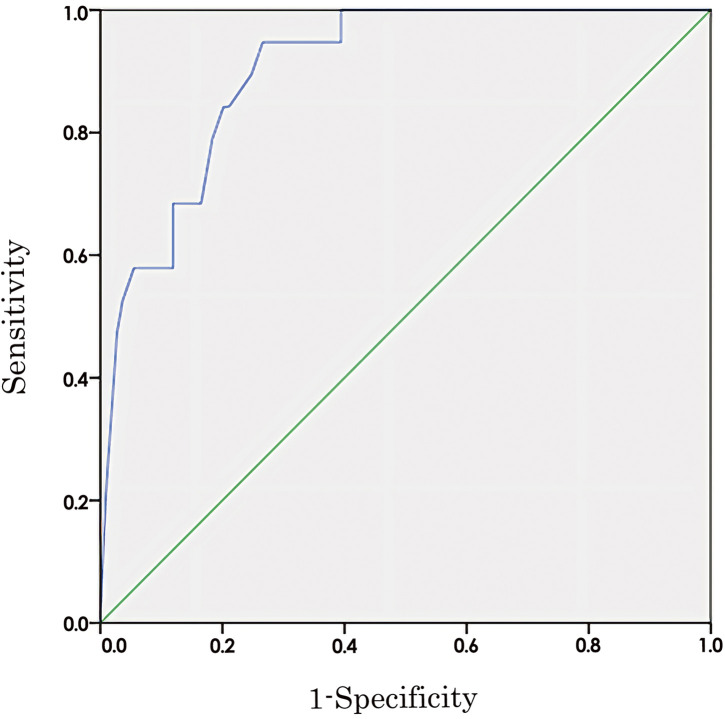
ROC curves of variables that predict discharge to home in the first week ROC, receiver operating characteristic

Figure 2 shows the ROC curve for the SIAS in predicting early home discharge. The area under the ROC curve of the SIAS was 0.90 (95% CI: 0.84–0.96), with an optimal cutoff value of 63.5 points. The sensitivity and specificity at this cutoff were 0.95 and 0.73, respectively.

## Discussion

In this study, we aimed to identify predictors of early home discharge in patients who underwent EVT for AIS. We found that only 14.8% of these patients were early discharged home, while the majority were transferred to other rehabilitation hospitals. Notably, the SIAS scores were significantly higher in patients who were early discharged home compared to those transferred. Logistic regression analysis, using the outcome of transfer as the dependent variable and first-week assessments as explanatory variables, identified SIAS and the FOIS as key predictors of early home discharge. The optimal cutoff value for SIAS was determined to be 63.5 points.

In our study, 84.2% of patients required transfer to rehabilitation hospitals or other facilities following EVT, in consistency with findings from D’Netto et al.^25)^, who reported that despite favorable treatment outcomes (68% with mTICI = 3), 56% of their cases still required transfer to rehabilitation hospitals, even though they could walk independently. The higher transfer rate observed in our study may reflect differences in grading scales and national healthcare systems, emphasizing that continued rehabilitation is often necessary for the majority of patients following EVT.

In this study, the SIAS emerged as a significant predictor of early home discharge following EVT. This finding underscores the importance of a thorough functional assessment and a strong overall SIAS score as prerequisites for the possibility of early home discharge. Supporting this, a previous systematic review and meta-analysis identified motor function at admission as a key factor associated with home discharge following stroke rehabilitation^12)^. Our findings align with these results. Additionally, Tsuji et al.^16)^ investigated the predictive validity of the SIAS and confirmed its utility in predicting stroke outcomes. Although the predictive validity of the SIAS in the acute phase had not been extensively studied before, our research demonstrates its effectiveness in this context. Thus, assessing total functional impairment using the SIAS in patients with AIS post-EVT may be a valuable approach for predicting discharge outcomes.

Dysphagia is a common functional impairment following stroke, affecting approximately 24.3%–52.6% of patients^26,27)^. In this study, dysphagia was assessed using the FOIS, where FOIS levels 1 to 5 indicated varying degrees of dysphagia, with levels ≤5 classified as dysphagia^23)^. Previous studies have reported that dysphagia following EVT can range from 24% to 75.1%^25,28,29)^. Notably, in patients with AIS exhibiting a median NIHSS score of 15.0 post-EVT, dysphagia has been identified as an independent predictor of poor functional outcomes at 3 months^29)^. In this study, the presence or absence of dysphagia was identified as a significant predictor of home discharge from an acute care hospital. Dysphagia complicates dietary management, necessitating the preparation of food that is safe for swallowing. Failure to manage this can lead to stroke-associated pneumonia^30)^. Although no significant differences in social information were noted prior to the onset of the disease, the preparation of dysphagia-compatible food often requires assistance from co-residents and social services, making it challenging for individuals to manage independently. Therefore, dysphagia’s role as a predictor of discharge to home highlights the practical challenges and resource needs associated with early home discharge for patients with AIS undergoing EVT.

In this study, logistic regression analysis was performed using assessments conducted 1 week after EVT treatment. The analysis identified the presence or absence of SIAS and dysphagia as factors associated with early home discharge. However, given the retrospective and observational nature of the study, the results should be interpreted with caution. While studies focusing on predicting outcomes specifically for AIS treated with EVT are limited, the strength of this study lies in its ability to identify and explore cases where early discharge to home is feasible. The identified cutoff value of 63.5 points for the SIAS provides predictive utility similar to that observed in general internal medicine, suggesting its potential applicability in clinical practice. Nonetheless, as comprehensive indicators are commonly used to predict discharge destinations, caution is warranted when generalizing these findings.

However, this study has several limitations. First, it is a single-center study with a relatively small sample size, which necessitates further validation with a larger cohort and across different centers. Second, dysphagia was defined using the FOIS, but it remains unclear whether patients had dysphagia prior to the onset of the disease. Further validation is needed to address this issue. Third, the definition of sICH has not been adequately standardized, despite validation of its predictors^11)^. Fourth, time from onset to EVT was significantly different in the 2-group comparison but was not a significant associated factor in the logistic regression analysis. The two-group comparison showed that the transfer group had a shorter time from onset to EVT. This may include cases of wake-up with unknown onset times. Finally, alternative assessment methods, such as the Fugl–Meyer Assessment^31)^, are also used to assess motor paralysis. Comparative validation of these indices remains an area for future research.

## Conclusion

We investigated predictive factors for early home discharge in patients with AIS who underwent EVT. Comprehensive functional disability index using the SIAS and the presence or absence of dysphagia using FOIS were predictive factors for early home discharge. Assessment of SIAS and swallowing function on day 7 in AIS patients undergoing EVT may be an important factor in predicting early discharge. Therefore, when predicting early discharge, it is important for physiotherapists to pay attention to the assessment on day 7 after surgery.

## Funding

Not applicable.

## Conflicts of Interest

The authors have no conflicts of interest to disclose.
